# Why are Chinese workers so unhappy? A comparative cross-national analysis of job satisfaction, job expectations, and job attributes

**DOI:** 10.1371/journal.pone.0222715

**Published:** 2019-09-26

**Authors:** Xing Zhang, Micha Kaiser, Peng Nie, Alfonso Sousa-Poza

**Affiliations:** 1 College of Economics and Management, Northwest A&F University, Yang Ling, Shaanxi, China; 2 Institute for Health Care & Public Management, University of Hohenheim, Stuttgart, Baden-Wurttemberg, Germany; 3 School of Economics and Finance, Xi’an Jiaotong University, Xi’an, Shaanxi, China; 4 Institute of Labor Economics (IZA), Bonn, Germany; TED University, TURKEY

## Abstract

Using data from the 2015 International Social Survey Program (ISSP), this study conducts a multinational comparison of job satisfaction determinants and their drivers in 36 countries and regions, with particular attention to the reasons for relatively low job satisfaction among Chinese workers. Based on our results from a Blinder-Oaxaca decomposition analysis, we attribute a substantial portion of the job satisfaction differences between China and the other countries to different job attributes and expectations; in particular, to unmet job expectations for interesting work, high pay, and opportunities for advancement. We also note that, contrary to common belief, Chinese workers value similar attributes as Western workers but perceive their work conditions as very different from those in the West.

## Introduction

Both academics and HR specialists recognize that keeping workers happy is important for the organization because satisfied workers–being more productive [1–5], more loyal, and less likely to leave their jobs [6–11]–can positively impact company performance [12–15]. Not only does a comprehensive review study find a significant correlation between job satisfaction and job performance, especially in complex jobs [16], but other research associates low levels of job satisfaction with higher levels of absenteeism and counterproductive behavior [17, 18]. The extent to which workers consider their jobs satisfying is thus now a major focus in many disciplines, including psychology, economics, and management [19–24].

China offers a particularly interesting case study for job satisfaction because its Confucian-based work ethic of hard work, endurance, collectivism, and personal networks (g*uanxi*) expects Chinese employees to devote themselves to and take full responsibility for the job, work diligently, and generally align their values and goals with those of the organization [25]. Deeply rooted in this Confucianism is the construct of Chinese individual traditionality reflecting “a moral obligation to fulfill the normative expectations of a prescribed role to preserve social harmony and advance collective interests” [26]. Hence, for the traditionalist Chinese, self-identity is defined by role obligations within networks of dyadic social relationships, which may imply less relevance for the job satisfaction determinants that matter in Western countries. Yet one of the rare nationwide studies that examined job satisfaction in China [27], found not only that job satisfaction among employees aged 16–65 is relatively low–with only 46% explicitly satisfied–but also that worker expectations differ significantly from what their jobs actually provide. In particular, many jobs are less interesting than expected, which prevents workers from realizing their perceived potential, creating an expectations gap that is a strong determinant of job satisfaction. Unlike research for Western countries, however, their study finds no link between job satisfaction and turnover, an outcome they attribute to China’s unique Confucian-based work ethic.

Despite this clear documentation of relatively low job satisfaction in China, however, few extant studies systematically and comprehensively compare such satisfaction with that in other countries. To begin filling this void, this present analysis draws on data for 36 countries, including China, from one of the most comprehensive cross-national surveys on job satisfaction ever conducted. One unique aspect of this survey is that it collects information not only on actual job characteristics but also on worker perceptions of what an ideal job should entail. As pointed out by Locke, “Job satisfaction is the pleasurable emotional state resulting from the appraisal of one's job as achieving or facilitating the achievement of one's job values. Job dissatisfaction is the unpleasurable emotional state resulting from the appraisal of one's job as frustrating or blocking the attainment of one's job values or as entailing disvalues. Job satisfaction and dissatisfaction are a function of the perceived relationship between what one wants from one's job and what one perceives it as offering or entailing” [23]. It is thus this expectations gap which is fundamentally driving job satisfaction. Unfortunately, much of the job satisfaction literature focuses solely on job attributes, and not on how these are evaluated. Hence, in addition to decomposing job satisfaction differences between China and other country clusters (using the Blinder-Oaxaca method), we are also able to determine the extent to which work-related expectations are being met and how they relate to low job satisfaction, thereby helping to explain its drivers. In doing so, we also provide additional evidence to a previous study [27] that found lower job satisfaction in China, particularly in relation to Western countries.

Identifying the determinants of job satisfaction in China and understanding differences in these determinants to other countries is important from a management perspective. Western countries are investing billions in China and many multinational companies have set up major manufacturing and distribution facilities in China. These companies not only employ very many Chinese workers, they are also frequently managed by international teams that often apply Western HR concepts. Yet considering China’s very different social and cultural background, it is important to assess Chinese employees’ responses to such Western HR concepts. In this paper we provide evidence on what Chinese workers value in a job and how these values differ to workers in other countries. This is an important precondition for a deeper understanding of the effectiveness of HR policies in China.

## Previous research

Despite a large body of literature on the determinants of job satisfaction [6, 19–22, 24, 28–35], 2021the research for China is restricted mostly to particular geographic areas [36–43] or specific occupations, including teachers [44–46], physicians [47, 48], nurses [49–53], civil servants [54], and migrant workers [55, 56]. To our knowledge, only four studies focus broadly on all employees across the nation. The first, based on 2002 China Mainland Marketing Research Company data for 8,200 employees in 32 cities, identifies age, education, occupation, and personal income as the main determinants of job satisfaction [57], while the second [58], drawing on 2008 Chinese General Social Survey (CGSS) data for urban locals, first-generation migrants (born before 1980), and new-generation migrants (born 1980 or thereafter) pinpoints income and education. The third study, based on 2006 CGSS data, not only identifies lower job satisfaction among female employees than among male employees, but positively associates job satisfaction with higher levels of education and communist party membership [59]. It also demonstrates, however, that job tenure, job security, earnings, promotion, and having a physically demanding job are significantly and positively correlated with job satisfaction for both sexes [59]. The final study [27] is already referenced, which uses a combination of 2012 China Labor-Force Dynamic Survey (CLDS) data and 2012–2014 China Family Panel Studies (CFPS) data to document the relatively low Chinese worker job satisfaction and significant job expectation gap, which reduces worker ability to reach perceived potential and greatly determines (low) job satisfaction.

Although the number of cross-national analyses in this area is limited, one study [24], using data from the 1997 International Social Survey Program (ISSP), document that 79.7% of employees in 21 countries report being fairly satisfied or satisfied with their job. Such satisfaction is significantly impacted by work-role inputs and outputs, with having an interesting job and good relations with management being the major determinants. Subsequent work [35], based on data from phase two of the Collaborative International Study of Managerial Stress (CISMS 2), reports a significantly lower average job satisfaction for their Asian country cluster (7.9) than for their Anglo Saxon (9.6), Eastern European (9.2), and Latin American (9.6) country clusters, with a 2-item job satisfaction measure ranging from 2 to 12. Their results support the assumption that the linkages between work demands and work interference with family (WIF) and between WIF and both job satisfaction and turnover intentions are stronger in individualistic Anglo-Saxon countries than in more collectivistic world regions, including Asia, Eastern Europe, and Latin America.

Other research focuses either on specific subpopulations of the workforce or particular aspects, such as skills and benefits. For instance, one of these previous studies [31], using 1994–2001 European Community Household Panel (ECHP) data, demonstrate that self-employed workers are more likely than paid employees to be satisfied with their present job type but less likely to be satisfied with the corresponding job security. More recent work [60] uses 2005 ISSP data for 32 countries, shows that women and mothers occupy more satisfying jobs in countries with more extensive workplace flexibility. As regards job skills, another more recent study [61], using Programme for the International Assessment of Adult Competencies (PIACC) data for 17 OECD countries, reports that the impact of labor mismatches on job satisfaction is generally better explained by skills mismatch, although educational mismatches have a greater effect on wages. Lastly, drawing on Global Entrepreneurship Monitor (GEM) data, some literature reveals that although entrepreneurial innovation benefits the job satisfaction, work-family balance, and life satisfaction of entrepreneurs globally, in China, it benefits only satisfaction with work-family balance and life–not job satisfaction [62].

As this brief review underscores, with the notable exception of the recent study [27] mentioned above, not only are representative investigations into job satisfaction determinants in China rare, but, more important for our study, so are cross-national studies, especially ones addressing China’s relatively low level of employee job satisfaction. We are also unaware of studies which explicitly assess job attributes, that is the extent to which certain attributes are present and also cherished. Hence, to expand understanding of this issue, we decompose the job satisfaction differences between China and several other country clusters to assess the universality and generalizability of particular determinants of job satisfaction and, importantly, the extent to which differing expectations about a job explain China’s job satisfaction level.

## Data and methods

Data: Our analysis is based on data from the 2015 ISSP, an ongoing collaborative administration of annual cross-national surveys on topics important for the social sciences. Begun in 1984 with four founding members, the program now includes about 50 member countries from all over the world. Whereas three previous surveys (1989, 1997, and 2005) included a section on work orientation and collected data on job attitudes and job characteristics, China did not participate in this module until 2015. Drawing on this 2015 data set, we analyze a sample of 17,938 individuals in 36 countries and regions: Austria, Belgium, Chile, China, Croatia, Czech Republic, Denmark, Estonia, Finland, France, Georgia, Germany, Great Britain, Hungary, Iceland, India, Israel, Japan, Latvia, Lithuania, Mexico, New Zealand, Norway, Philippines, Poland, Russia, Slovakia, Slovenia, South Africa, Spain, Suriname, Sweden, Switzerland, Taiwan (province of China), and the United States. The ISSP survey is usually included in other large surveys (with only a handful of countries conducting single surveys). In most countries, face-to-face interviews with multi-stage sampling were conducted (in some countries such as Poland questionnaires were self-completed with interviewer involvement). All surveys were conducted in the national language(s). Translations were evaluated by experts and, in some countries, by back-translation. Each country used a specific stratification strategy, with China, for example, using education, GDP per capita, and urbanization [63]. Our final sample excludes all self-employed workers to cover only those currently in paid employment (see S1 Table for summary statistics for the entire sample and for China only). Note that the sampling procedure differs somewhat in each country, and the main sampling quotas (i.e., age, gender and education) are based on the composition of the whole population of a respective country, not just on the labor force.

Defining clusters of countries: When comparing China’s job satisfaction with the job satisfaction in other nations, one must decide on how to construct a comparison group. Several options are possible, including country-by-country comparisons, comparing China with “the rest of the world”, or grouping countries according to some characteristics. In order to take account of the heterogeneity in job characteristics and job expectations among countries, and yet to provide insights in a summarized and tractable way, we have opted for clustering countries according to a few economic and sociodemographic characteristics. We partition the remaining 35 countries and regions into 3 clusters by using the *k-means* clustering algorithm [64]. The algorithm begins by assigning a random number to each observation. These serve as initial cluster assignments for the observations. For each of the clusters the algorithm then computes the clusters’ centroids (vector of the clusters’ variable means) and assigns each observation to the cluster whose centroid is closest (where closest is defined using Euclidean distance). This process continues until assignments do not change anymore [65]. To obtain a valid assignment of each country to a specific cluster, we run the algorithm 400 times, with each iteration using different cluster assignments at the beginning. We base our cluster analysis on the country specific mean values of certain variables within the data set, namely working hours, income in US-Dollars, age, years of education, family size and marital status. The obtained clusters are the following:

- Cluster 1: Chile, Croatia, Czech Republic, Estonia, Georgia, Hungary, India, Latvia, Lithuania, Mexico, Philippines, Poland, Russia, Slovak Republic, Slovenia, South Africa, Spain, Suriname, Taiwan (province of China).
- Cluster 2: Australia, Denmark, Iceland, Norway, Switzerland.
- Cluster 3: Austria, Belgium, Finland, France, Germany, Israel, Japan, New Zealand, Sweden, Great Britain, United States.

The largest Cluster 1 includes all Eastern European countries, Russia, the Baltic states, as well as a few other countries from Asia, Western Europe and South America. Cluster 2 primarily captures the Nordic countries, as well as Switzerland and Australia. Cluster 3 is made up of primarily Western European countries and the United States. Summary statistics for each cluster are presented in S2 Table. As can be seen in the summary statistics, Cluster 1 is characterized by a higher number of average working hours, as well as a larger family size on average compared to Clusters 2 and 3. The average monthly income and the educational level are substantial lower in Cluster 1 compared to the other two clusters. The mean age in Cluster 2 is the highest among all three clusters. Cluster 2 also exhibits the most educated and (in terms of income) wealthiest population, yet having the lowest amount of weekly working hours. Only minor differences among Clusters 2 and 3 exist with regards to the average marital status and family size of the population.

Although our objective is to construct homogenous clusters based on economic and sociodemographic variables, we also conducted an analysis using the GLOBE country classification, which groups nations by cultural characteristics; however, the main conclusions remain unchanged. In a further sensitivity analysis, we also clustered countries according to their Human Development Index which is a composite index of life expectancy, education, and per capita income at the country level. The main conclusions of this paper, however, remain unchanged.

Country differences: Although the 7-point scaling of our job satisfaction measure might suggest a latent variable estimation approach as the most appropriate, because the bias introduced by an OLS analysis is relatively small [66], we employ the standard OLS regression method applied in the majority of SWB studies [67]. Hence, to pinpoint the differences among countries, we estimate a series of linear regressions (OLS) of the following form:

$${JS}_{i}=\beta_{0}+\beta_{1}C_{i}+\beta_{2}S_{i}+\beta_{3}A_{i}+\beta_{4}E_{i}+\varepsilon_{i}$$ (1)

where *JS_i_* denotes job satisfaction of individual *i*, *C_i_* is the country dummy variable (with Germany as the reference group because its job satisfaction mean falls roughly mid sample). *S_i_*, *A_i_* and *E_i_* represent socioeconomic and demographic characteristics, work attributes, and work expectations, respectively, while *ε_i_* is the error term. Job satisfaction is measured by the question, “How satisfied are you in your (main) job?” with responses measured on a 7-point scale from “1 = completely satisfied” to “7 = completely dissatisfied.” For convenience of interpretation, we recode the values so that 7 reflects the highest job satisfaction and 1 the lowest. This job satisfaction measure, although based only a single-item, is empirically documented to be acceptable [68].

Work attributes. Based on prior literature and data availability, we use seven variables to capture work attributes:

- Hours worked per week (including overtime).
- Work time conditions: Based on the response to “Which statement best describes how your work hours are decided? 1 = fixed time, 2 = decide with limits, and 3 = free to decide,” we create two dummy variables for 1 and 3, with 2 as the reference.
- Daily work organization: Using responses to “How is your daily work organized? 1 = not free to decide, 2 = with certain limits, 3 = free to decide,” we again formulate two dummy variables for 1 and 3 with 2 as the reference.
- Work schedules: Based on responses to “Which statement best describes your usual working schedule in your main job? 1 = decided by the employer, 2 = scheduled with changes, 3 = regular schedule,” we generate dummies for 1 and 3, with 2 as the reference.
- Employer-employee relations: From responses to the question, “In general, how would you describe relations at your workplace between management and employees? 1 = very bad, 2 = quite bad, 3 = neither good nor bad, 4 = quite good, 5 = very good,” we derive a 3 category coding of 1 = bad, 2 = neither good nor bad, 3 = good, from which we create dummies for 1 and 3, with 2 as the reference.
- Relations between colleagues: We similarly recode the responses to “In general, how would you describe relations at your workplace between workmates/colleagues? 1 = quite bad, 2 = very bad, 3 = neither good nor bad, 4 = quite good, 5 = very good” as 1 = bad, 2 = neither good nor bad, 3 = good, and generate the two dummies for 1 and 3, with 2 as a reference.
- Work pressure: From responses to “How often do you find your work stressful? 1 = never, 2 = hardly ever, 3 = sometimes, 4 = often, 5 = always,” we derive a 4-category recoding of 1 = never, 2 = sometimes, 3 = often, 4 = always, and define three dummy variables for 1, 3, and 4, with 2 as the reference.

Work expectations. We assess work expectations based on the discrepancy between personal importance (what is wanted) and perceived outcome (what is obtained) of a given work facet; namely, job security, income, job interest, promotion opportunities, work independence, usefulness to society, helping others, and contact with other people. We derive our variables from responses to related survey questions, all measured on a 5-point scale. Specifically, individuals are first asked to assess the importance of these job attributes on a scale ranging from 1–5 (from 1 = not important at all to 5 = very important). Thus, for example, the question related to job security is formulated as follows: “How important is job security?” Individuals are then asked to assess their current job using the same scale (from 1 = strongly disagree to 5 = strongly agree). In the case of job security, the question is as follows: “How much do you agree or disagree that it applies to your job: my job is secure”. We then calculate work expectations by subtracting the value assigned a specific job characteristic’s importance from the value depicting its actual presence in the job, thereby capturing unmet expectations with variables valued from -4 to 4. Clearly, a negative value has a conceptually different meaning than a positive value. More specifically, a negative value indicates that a characteristic of the current job is more pronounced than the importance giving to it, whereas a positive value is more akin to unmet expectations. In order to take these different concepts into account, our regressions include dummy variables for each characteristic that are equal to one if the difference is negative or zero, and zero otherwise. It should be noted that, for most job characteristics, values are seldom negative (less than 10% of observations). Only with regards to “contact with other people” do we have 36% negative values, indicating that about a third of workers have contact with other people, but do not value this characteristic highly.

### Socioeconomic and demographic variables

Our socioeconomic and demographic controls are those usually included in job satisfaction regressions [6, 24]; namely, age, gender (a dummy equal to 1 for males, and 0 for females), education (measured by years of schooling), and family size. Marital status is recoded into three dummies for married, divorced, and widowed (with single as the reference). To capture personal income, we convert income data into a categorical variable based on a 3-point scale from 1 = low to 3 = high, with the top and bottom 25% of personal income defining a country’s high and low levels, respectively, and the middle 50% designating the mid-level (with low as the reference category).

Decomposing job satisfaction differences: To identify which specific determinants account for the job satisfaction gap between China and other countries, we employ a mean-based Blinder-Oaxaca (BO) decomposition [69, 70] that assumes a linear and additive nexus between job satisfaction and a given set of characteristics. One advantage of BO decomposition over regression analysis is that it quantifies the contribution of specific factors that account for job satisfaction differences between China and a specific cluster. In our case, the total difference in mean job satisfaction can be decomposed as follows:

$${\bar{Y}}^{C}-{\bar{Y}}^{Cl}=({\bar{X}}^{C}-{\bar{X}}^{Cl}){\hat{\beta }}^{C}+{\bar{X}}^{Cl}({\hat{\beta }}^{C}-{\hat{\beta }}^{Cl})$$ (2)

where ${\bar{X}}^{i}$ is a vector of the average values of the independent variables and ${\hat{\beta }}^{i}$ is a vector of the coefficient estimates for China (denoted by *C*) and a specific cluster (denoted by *Cl*). In Eq (2), the first (explained) term on the right indicates the contribution of a difference in the distribution of determinant *X*, while the second (unexplained) term refers to the part attributable to a difference in the determinants’ effects [71]. The second term thus captures all the potential effects of differences in unobservables. In keeping with the majority of previous research using decomposition [72], we focus on the explained terms and their disaggregated contribution for individual covariates, with a variable’s contribution given by the average change in the function if that variable changes while all other variables remain the same. It is important to note that this decomposition does not reveal causal relations but rather decomposes the change in job satisfaction between China and some cluster by assessing the change in the observables associated with job satisfaction. These are merely associations and cannot infer the direction of a relationship. Thus, it is conceivable that certain expectations not only affect job satisfaction, but that job satisfaction may in turn affect expectations and the general assessment of a job. Hence, although we follow common practice in speaking of the “explained” part of the decomposition, we do so in full awareness that the analysis is not causal.

## Results

As Table 1 shows, average levels of job satisfaction range from 5.786 in Austria to 4.342 in Japan, with China, at 4.745, ranking second worst and substantially lower than the sample mean of 5.322. It is interesting to note that two Confucian Asia countries (Japan and China) are ranked last among 36 countries. Japan’s low average job satisfaction is quite impressive, being 0.403 points lower than that of China. The third Confucian Asia region, Taiwan, is also ranked quite low, yet its job satisfaction is a significant 0.430 points higher than that of China. Taiwan also has a higher average job satisfaction than non-Confucian countries such as France, Australia and Poland.

**Table 1:** Mean value of job satisfaction by country.

Country	Mean	Observations	Country	Mean	Observations
Austria	5.786	542	Slovenia	5.286	384
Mexico	5.689	305	India	5.281	153
Switzerland	5.661	608	Great Britain	5.279	700
Philippines	5.565	294	Croatia	5.264	447
Spain	5.504	557	Czech Republic	5.251	601
Israel	5.491	534	South Africa	5.225	569
Chile	5.471	427	Slovakia	5.224	441
Suriname	5.432	333	Sweden	5.203	526
Latvia	5.421	475	Belgium	5.197	877
Iceland	5.418	471	Estonia	5.196	562
United States	5.416	753	Taiwan, province of China	5.175	838
Norway	5.415	699	Hungary	5.129	388
Germany	5.406	751	Australia	5.080	411
Denmark	5.403	521	France	5.053	433
Finland	5.376	439	Lithuania	4.986	369
New Zealand	5.335	233	Poland	4.857	496
Russia	5.313	633	China	4.745	369
Georgia	5.289	273	Japan	4.342	526

Based on data from the 2015 ISSP.

**Fig 1:**
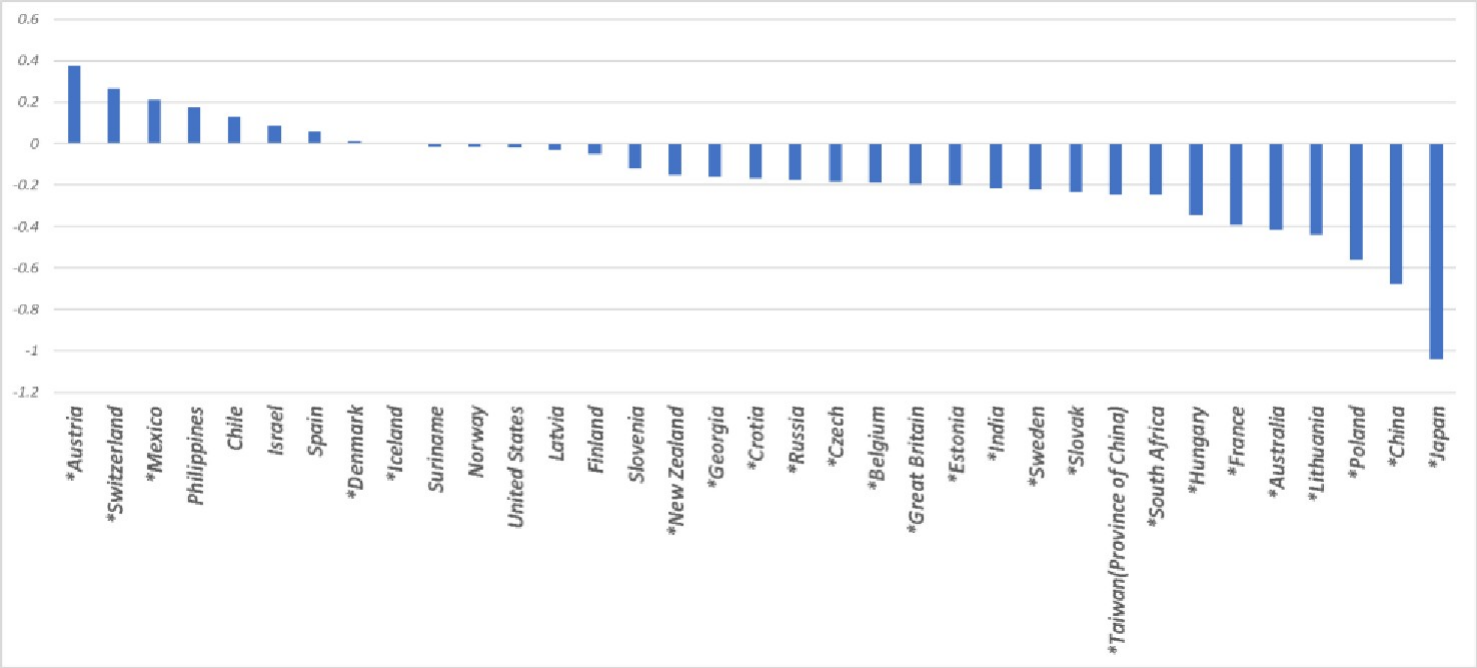
Job satisfaction coefficients by country: No control variables. Country coefficients based on 2015 ISSP data and calculated using linear job satisfaction regressions with no control variables and Germany as the reference. * p<0.05.

**Fig 2:**
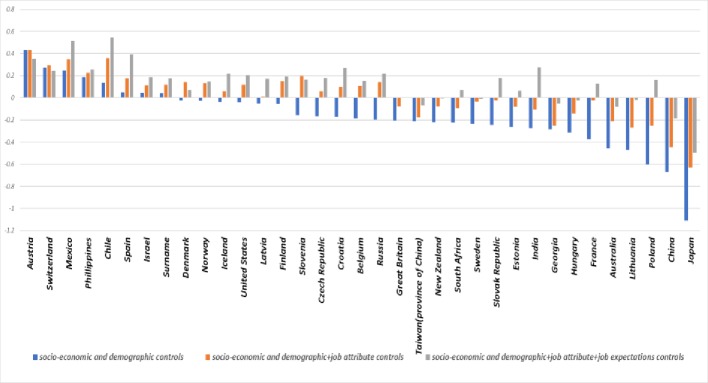
Job satisfaction coefficients by country: Control variables included. Country coefficients based on 2015 ISSP data and calculated using linear job satisfaction regressions with varying sets of control variables. Blue bars = country coefficients with socioeconomic and demographic controls; orange bars = country coefficients with socioeconomic, demographic, and job attribute controls; grey bars = country coefficients with socioeconomic, demographic, job attribute, and job expectation controls (see S3 Table for the full regression results).

Taking Germany as the reference country, our Fig 1 comparison shows its average job satisfaction to be 0.7 points higher than that of China. When we then run a series of regressions to assess the extent to which socioeconomic and demographic variables, job attributes, and job expectations affect this ranking, we find that the socioeconomic and demographic variables make little difference, but job attributes and job expectations substantially reduce the size of China’s coefficient (Fig 2). More specifically, whereas job attributes reduce the coefficient by 33%, adding in job expectations lowers it by 72%, meaning that these two sets of variables explain about two-thirds of the job satisfaction gap between Germany and China. The fact that even after we control for these three variable sets, China’s coefficient is a significant -0.19 indicating that cultural differences (probably in answering subjective questions on well-being) may play a certain role in job satisfaction differences among countries.

**Fig 3:**
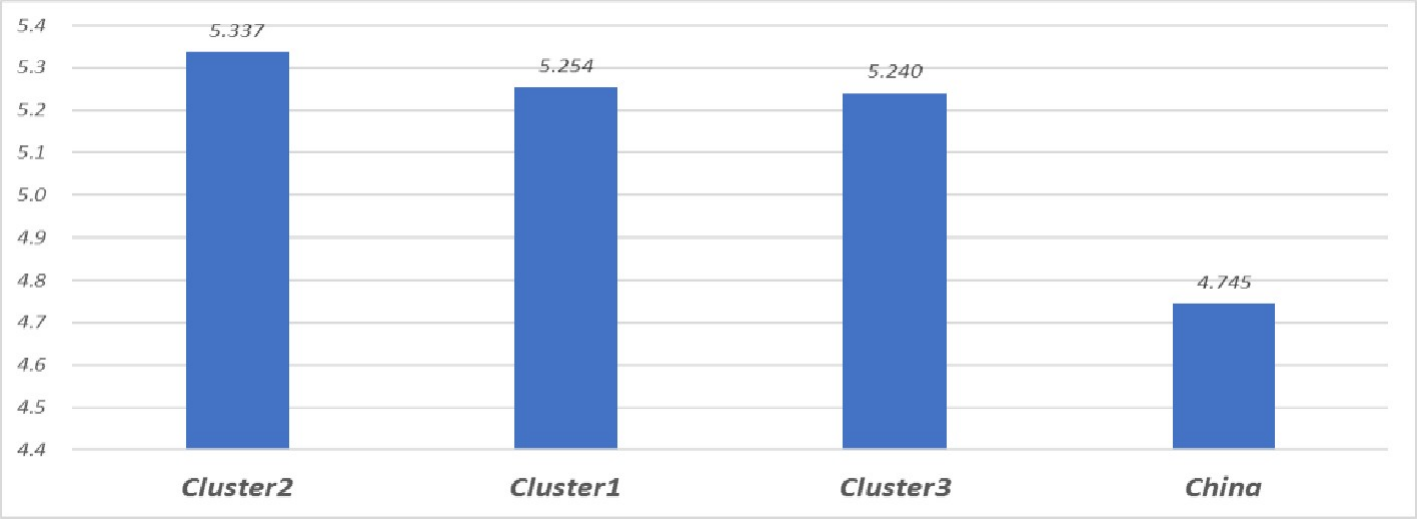
Mean job satisfaction by cluster, based on 2015 ISSP data. Cluster 1: Chile, Taiwan (province of China), Croatia, Czech Republic, Estonia, Georgia, Hungary, India, Latvia, Lithuania, Mexico, Philippines, Poland, Russia, Slovak Republic, Slovenia, South Africa, Spain, Suriname. Cluster 2: Australia, Denmark, Iceland, Norway, Switzerland; Cluster 3: Austria, Belgium, Finland, France, Germany, Israel, Japan, New Zealand, Sweden, Great Britain, United States.

For a more in-depth explanation of China’s markedly low levels of job satisfaction, we decompose the satisfaction differences between China and our three country clusters. The differences in average job satisfaction are presented in Fig 3 and they reveal a substantially lower average for China, but relatively small differences among the three clusters. The results of the BO decomposition are presented in Table 2 and they show that 30%–46% of the job satisfaction differences between China and the three clusters are associated with differences in socioeconomic and demographic characteristics, job attributes, and job expectations. More specifically, 31% of the gap between China and Cluster 1 is associated with differences in job attributes and job expectations, while 44% of that between China and Cluster 2 and Cluster 3 is associated with differences in job expectations. Thus, unmet job expectations appear to be a major driver of China’s low levels of job satisfaction.

**Table 2:** Blinder-Oaxaca decomposition of job satisfaction differences between China and our country clusters.

	*Cluster 1*	*Contribution* *(%)*	*Cluster 2*	*Contribution* *(%)*	*Cluster 3*	*Contribution* *(%)*
Total difference	-0.531***		-0.672***		-0.524***	
	(0.052)		(0.055)		(0.053)	
Unexplained	-0.373***	70	-0.366***	54	-0.335***	63
	(0.049)		(0.064)		(0.056)	
Explained	-0.157***	30	-0.306***	46	-0.189***	37
	(0.036)		(0.053)		(0.046)	
Explained part						
Sociodemographics	0.008	-1	0.021	-3	0.038**	-7
	(0.012)		(0.019)		(0.018)	
Job attributes	-0.064***	12	-0.030	5	0.002	-0.4
	(0.021)		(0.032)		(0.028)	
Job expectations	-0.101***	19	-0.298***	44	-0.228***	44
	(0.023)		(0.035)		(0.029)	
Observations	8,914		3,079		6,683	

Estimates based on 2015 ISSP data. Standard errors in parentheses.

* p<0.1

** p<0.05

*** p<0.01.

**Fig 4:**
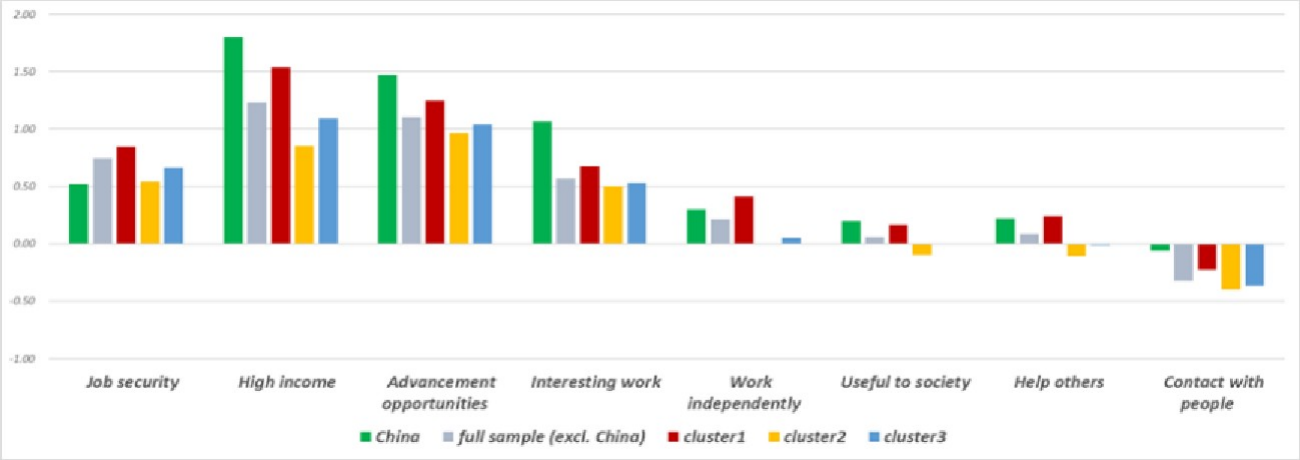
Expectations gap by cluster. The graph, based on 2015 ISSP data, shows the difference between what workers consider important in a job and what they report having in a job. The full sample excludes China.

We summarize the five variables that account for most of the job satisfaction differences between China and the three clusters in Table 3, and graph the job expectations gap in Fig 4. What is evident from both graphics is that unmet expectations for an interesting job is by far the most important variable, accounting for 19–34% of the job satisfaction difference (Table 3). In fact, as can be seen in the descriptive statistics in S1 and S2 Figs, although about 82% of the Chinese workers believe that having an interesting job is important, compared to 91%, 95%, and 93% or workers in Clusters 1–3, respectively; only 36% consider their jobs interesting, compared to 65%, 78%, and 74% in Clusters 1–3, respectively. Unmet expectations for income also matter, with about 94% of Chinese workers thinking it important to earn a high income, versus only about 90%, 70%, and 77% of workers in Clusters 1–3, respectively (S1 Fig). Again, however, only about 23% of the Chinese sample agrees that the current position offers a high income, compared with 34% and 30% for Clusters 2 and 3, respectively (S2 Fig). Unmet expectations for income thus appear to have a greater influence on job satisfaction level in China than in more Western economies.

**Table 3:** Top five contributors to the job satisfaction differences with China.

Cluster 1	(%)	Cluster 2	(%)	Cluster 3	(%)
Interesting job (expectations)	19	Interesting job (expectations)	32	Interesting job (expectations)	34
High income (expectations)	8	High income (expectations)	8	High income (expectations)	10
Daily work organization	6	Relations between colleagues	5	Daily work organization	9
Employer-employee relations	4	Daily work organization	5	Relations between colleagues	3
Relations between colleagues	2	Advancement (expectations)	4	Working hour condition	1

Based on a Blinder-Oaxaca decomposition of 2015 ISSP data, with percentages based on the percent of total difference in job satisfaction (see S3 Table column (3) for regression results)

Another aspect that contributes to job satisfaction is the freedom to organize one’s own daily work, which only 15% of Chinese workers report having, compared to 27%, 25%, and 26% in Clusters 1–3, respectively (S3 Fig). Even the good relationships with colleagues, which 78% of the Chinese workers admit to having, is significantly lower than the 85%, 90%, and 86% reported by workers in Clusters 1–3, respectively (S3 Fig). Good relations with the employer (at 66%) are also slightly lower in China than in other countries (74%, 73%, and 72% for Clusters 1–3, respectively; see S3 Fig). A final contributing factor is opportunity for advancement, considered important by 77% of Chinese workers but an expectation that only 19% believe is being met in their current position. In Clusters 1–3, in contrast, the percentages of workers who believe their jobs offer good advancement opportunities are 30%, 26%, and 28%, respectively, reflecting considerable expectation gaps but none so large as in China (S2 Fig).

## Discussion and conclusions

Given the scarcity of cross-national job satisfaction research that includes China, this present analysis of 2015 ISSP data is most probably the first comprehensive comparison of job satisfaction in China with that in a large sample of other countries. As anticipated by a previous study [27], our results confirm that job satisfaction in China is substantially lower than in most of the other countries studied, ranking second to last of 36. By clustering these countries into three homogeneous groups based on observable economic and sociodemographic characteristics, we are able to identify several reasons for this relatively low job satisfaction, three of which are particularly important.

The most notable driver of low job satisfaction across all comparison clusters is unmet expectations for how interesting a job should be. Although Chinese workers’ expectations for this attribute are similar to those of workers in other countries, they consider their jobs substantially less interesting. This finding supports the claim that a large proportion of jobs fail to satisfy worker interests [27]. One possible cause of this proliferation may be the vertical relations (i.e., rigid top-down hierarchy and paternalism) that still dominate Chinese business organizations, which may hamper workers’ ability to organize their own daily activities and stiffen self-initiative, which would make the job less interesting. At the same time, however, as S1 Table shows, the share of workers who value the importance of job security (95%) and high income (94%) is larger than the share that values job interest (82%). Thus, Chinese workers, unlike those in our country clusters, value job security and a good income more than having an interesting job, implying that they would rather sacrifice personal interest for a well-paid, guaranteed position. It is therefore not surprising that most young people attending college in China today choose their majors based mainly on future job security and income considerations, and less on intrinsic interest [73].

This importance that Chinese workers place on a well-paying job, which is higher than in most Western countries, generates a second driver of dissatisfaction, the tendency for workers to judge their own current wage as inadequate. In fact, according to CLDS data, the financial aspect has become the most important job characteristic in China [27], an observation that totally contradicts the widely held belief that earnings are less of an intrinsic motivator in Confucian societies. Of course, the per capita annual disposable income of residents in China is approximately 21,966yuan (equivalent to 3,527US$) in 2015, which is indeed lower than in most developed countries [74]. Nonetheless, individuals in all countries tend to assess their own incomes relative to those of their peers, which, given the dramatic increase in income disparity at various levels, could be contributing to the relatively low income satisfaction [75].

A third reason for job dissatisfaction identified by our decomposition analysis is the perception of relatively poor advancement opportunities, which is particularly pronounced in China, even though the amount of importance attributed to it differs little from that in other countries. Yet despite the importance attributed to advancement opportunities, only about 1 in 5 workers reports to having a job that actually offers such development perspectives (S2 Fig).

Even though these unmet expectations for an interesting well-paid job with attractive advancement opportunities can explain part of the job satisfaction gap between China and the other countries, however, a significant part remains unexplained. One briefly mentioned social aspect that should be emphasized here is that ways of responding to subjective questions on well-being may be culturally specific, making the Chinese workers’ low job satisfaction ranking no more than an artefact unassociated with actual job characteristics. Although we cannot refute this argument, which is seemingly supported by the considerable share of the satisfaction gap that our variables cannot explain, the markedly higher levels of job satisfaction reported by Taiwanese workers (a frequent proxy for the Chinese because of a common language and Confucian philosophy) is compulsive evidence against it. In fact, the difference in average job satisfaction between Taiwanese and Chinese workers of over 0.4 points is substantial (see Table 1).

Our results do make a useful contribution to the economic convergence or divergence literature [76–78], which examines whether, as economies develop, work attitudes converge irrespective of cultural context into a universal stance or whether underlying values and belief systems engender significant differences in employee expectations and attitudes. Our results provide ample evidence for convergence in that Chinese workers attribute similar importance to most job attributes as workers in other countries (S1 Fig). Interestingly, one of the few notable intercountry differences concerns income, with Chinese workers placing more importance on a well-paying job than their Western counterparts. Nonetheless, even though Chinese workers expect an interesting job, higher pay, and advancement opportunities, this expectation stems less from differing work attitudes or values than from perceptions of what the current job offers. This convergence is further underscored by the importance of developing good relations with coworkers, deemed as important in China as elsewhere despite a lower probability of Chinese workers having a job that allows such development. In fact, relationships with both colleagues and employers in China are not as good as those reported in all three clusters (S3 Fig), a somewhat surprising finding given the group orientation and participative decision-making encouraged by China’s collectivistic society.

Finally, cross-national studies such as ours are invaluable, “even indispensable,” to valid interpretation and generalizability of findings from research that, like the job satisfaction literature, tends to focus on Western countries and test assumptions specific to a single culture or society. Not only does cross-national investigation ensure that “social structural regularities are not mere particularities, the product of some limited set of historical or cultural or political circumstances,” it also forces researchers to “revise [their] interpretations to take account of cross-national differences and inconsistencies that could never be uncovered in single-nation research” (p. 77). [79]

## Supporting information

S1 Fig Importance of job attributes. (PDF)

S2 Fig Attributes of the current job. (PDF)

S3 Fig Work attributes of the current job. (PDF)

S1 Table Summary statistics for the entire sample and China. (PDF)

S2 Table Summary statistics for the three clusters. (PDF)

S3 Table Job satisfaction regressions. (PDF)
