# Supplementary material for: Why are Chinese workers so unhappy? A comparative cross-national analysis of job satisfaction, job expectations, and job attributes
Source: PLoS One. 2019 Sep 26;14(9):e0222715. doi: 10.1371/journal.pone.0222715 (PMC6762101; doi:10.1371/journal.pone.0222715)
Supplement: S1 Fig — (PDF) [file pone.0222715.s001.pdf]

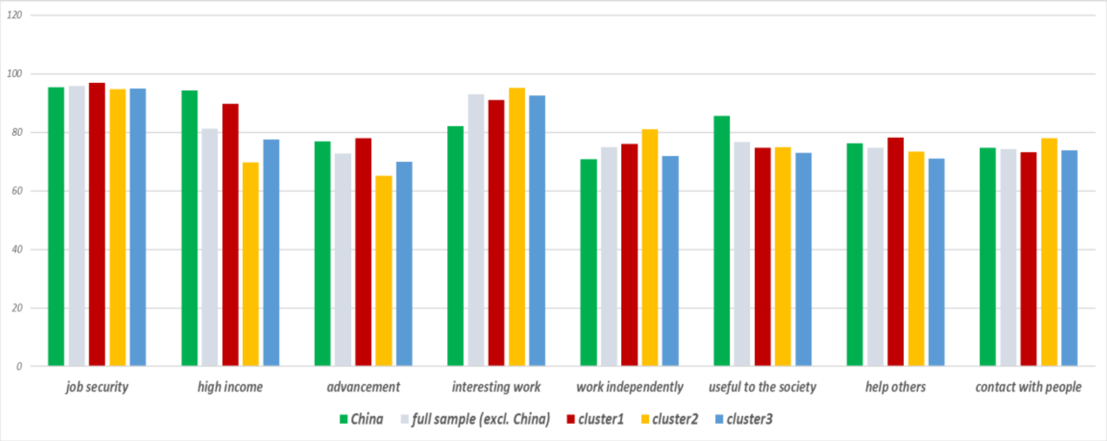

S1 Fig. Importance of job attributes.

The graph, based on 2015 ISSP data, shows the percentage of workers that consider the attribute to be important or very important. The full sample excludes China.
