# Supplementary material for: Why are Chinese workers so unhappy? A comparative cross-national analysis of job satisfaction, job expectations, and job attributes
Source: PLoS One. 2019 Sep 26;14(9):e0222715. doi: 10.1371/journal.pone.0222715 (PMC6762101; doi:10.1371/journal.pone.0222715)
Supplement: S2 Fig — (PDF) [file pone.0222715.s002.pdf]

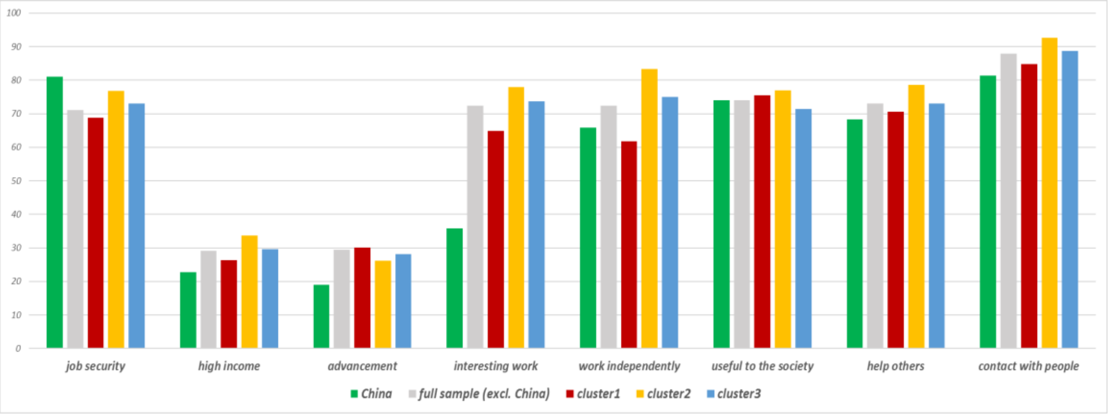

S2 Fig. Attributes of the current job.

The graph, based on 2015 ISSP data, shows the percentage of workers that agree or strongly agree that their current job has the attribute in question. The full sample excludes China.
