# Supplementary material for: Why are Chinese workers so unhappy? A comparative cross-national analysis of job satisfaction, job expectations, and job attributes
Source: PLoS One. 2019 Sep 26;14(9):e0222715. doi: 10.1371/journal.pone.0222715 (PMC6762101; doi:10.1371/journal.pone.0222715)
Supplement: S3 Fig — (PDF) [file pone.0222715.s003.pdf]

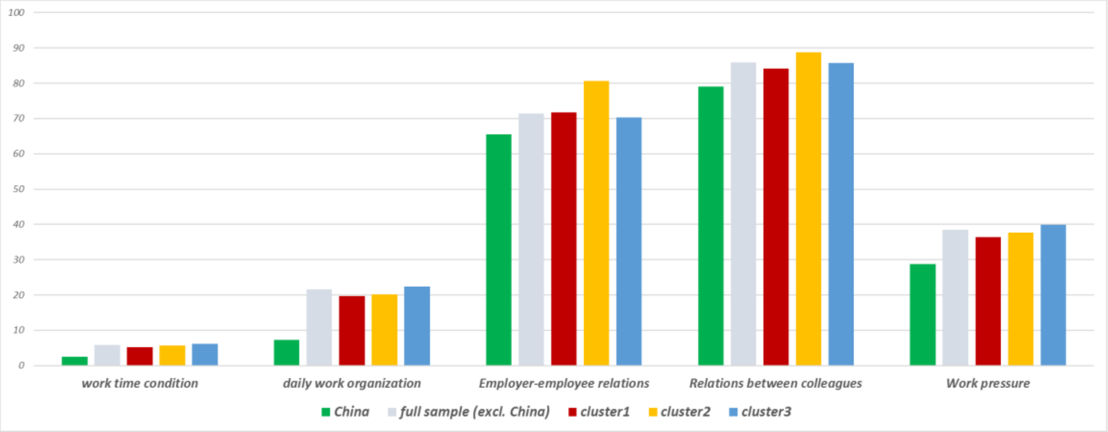

S3 Fig. Work attributes of the current job.

The graph, based on 2015 ISSP data, shows the percentage of workers that report a self-evaluation of each work attribute. Specifically, the results for work time condition and daily work organization refer to the "free to decide" category, those for employer-employee relations and relations between colleagues refer to the "quite good" and "very good" categories, and those for work pressure refer to the "often" and "always" categories. The full sample excludes China.
