# Supplementary material for: Why are Chinese workers so unhappy? A comparative cross-national analysis of job satisfaction, job expectations, and job attributes
Source: PLoS One. 2019 Sep 26;14(9):e0222715. doi: 10.1371/journal.pone.0222715 (PMC6762101; doi:10.1371/journal.pone.0222715)
Supplement: S1 Table — (PDF) [file pone.0222715.s004.pdf]

S1 Table. Summary statistics for the entire sample and China

	Entire sample[a]		China[b]	
	**Mean**	**Standard deviation**	**Mean**	**Standard deviation**
**Job satisfaction**	5.322	1.155	4.745	0.978
Completely dissatisfied	0.008	0.086	0.008	0.090
Very dissatisfied	0.015	0.123	0.005	0.074
Fairly dissatisfied	0.049	0.216	0.100	0.301
Neither satisfied nor dissatisfied	0.110	0.313	0.198	0.399
Fairly satisfied	0.383	0.486	0.520	0.500
Very satisfied	0.299	0.458	0.144	0.351
Completely satisfied	0.135	0.341	0.024	0.154
**Sociodemographic variables**				
Age	42.238	12.446	38.783	11.193
Education (years of schooling)	13.545	3.330	11.837	4.181
Male	0.488	0.500	0.485	0.500
Marital status: married	0.582	0.493	0.805	0.397
Marital status: divorced	0.094	0.292	0.022	0.146
Marital status: widowed	0.024	0.154	0.014	0.116
Marital status: single	0.300	0.458	0.160	0.367
Family size	3.066	1.461	2.940	1.364
Income: low	0.123	0.329	0.081	0.274
Income: middle	0.459	0.498	0.466	0.500
Income: high	0.418	0.493	0.453	0.498
**Work attributes**				
Hours worked weekly	40.461	11.989	47.482	15.517
Work time conditions				
Fixed time	0.599	0.490	0.759	0.428
With limits	0.344	0.475	0.217	0.413
Free to decide	0.057	0.232	0.024	0.154
Daily work organization				
Can't decide	0.316	0.465	0.455	0.499
With limits	0.472	0.499	0.471	0.500
Free to decide	0.212	0.409	0.073	0.261
Work schedules				
Decide by the employer	0.099	0.298	0.106	0.308
Schedule with changes	0.149	0.356	0.198	0.399

gular schedule	0.752	0.432	0.696	0.460
Employer-employee relations				
Very bad	0.019	0.137	0.000	0.000
Quite bad	0.054	0.227	0.024	0.154
Neither good nor bad	0.213	0.410	0.320	0.467
Quite good	0.470	0.499	0.553	0.498
Very good	0.243	0.429	0.101	0.304
Relations between colleagues				
Very bad	0.006	0.074	0.000	0.000
Quite bad	0.019	0.135	0.014	0.116
Neither good nor bad	0.118	0.323	0.195	0.397
Quite good	0.500	0.500	0.640	0.481
Very good	0.358	0.459	0.152	0.359
Work pressure				
Never	0.208	0.406	0.247	0.432
Sometimes	0.409	0.492	0.466	0.500
Often	0.273	0.446	0.225	0.418
Always	0.109	0.312	0.062	0.242
**Personal importance (what is wanted)**				
Job security				
Not important at all	0.002	0.042	0.003	0.052
Not important	0.011	0.105	0.022	0.146
Neither nor	0.029	0.168	0.022	0.246
Important	0.338	0.473	0.528	0.500
Very important	0.620	0.485	0.425	0.495
High income				
Not important at all	0.002	0.049	0.003	0.052
Not important	0.038	0.191	0.011	0.104
Neither nor	0.144	0.351	0.043	0.204
Important	0.513	0.500	0.528	0.500
Very important	0.303	0.459	0.415	0.493
Advancement opportunities				
Not important at all	0.013	0.111	0.003	0.052
Not important	0.078	0.268	0.041	0.198
Neither nor	0.181	0.385	0.187	0.390
Important	0.447	0.497	0.569	0.496
Very important	0.282	0.450	0.201	0.401

Interesting work				
Not important at all	0.002	0.049	0.000	0.000
Not important	0.014	0.116	0.043	0.204
Neither nor	0.057	0.232	0.136	0.343
Important	0.431	0.495	0.599	0.491
Very important	0.431	0.495	0.599	0.491
Work independently				
Not important at all	0.010	0.097	0.002	0.052
Not important	0.065	0.247	0.046	0.210
Neither nor	0.176	0.381	0.244	0.430
Important	0.456	0.498	0.547	0.498
Very important	0.293	0.456	0.160	0.367
Useful to society				
Not important at all	0.011	0.106	0.003	0.052
Not important	0.043	0.202	0.022	0.146
Neither nor	0.178	0.382	0.119	0.325
Important	0.477	0.500	0.607	0.489
Very important	0.291	0.454	0.249	0.433
Help others				
Not important at all	0.009	0.096	0.000	0.000
Not important=2	0.046	0.209	0.033	0.178
Neither nor	0.197	0.397	0.206	0.405
Important	0.473	0.499	0.599	0.491
Very important	0.275	0.447	0.163	0.370
Contact with people				
Not important at all	0.016	0.124	0.005	0.074
Not important	0.066	0.247	0.054	0.227
Neither nor	0.177	0.381	0.192	0.395
Important	0.457	0.498	0.588	0.493
Very important	0.286	0.452	0.160	0.367
**Perceived outcomes (what is obtained)**				
Job security				
Strongly disagree	0.035	0.183	0.024	0.154
Disagree	0.109	0.312	0.119	0.325
Neither nor	0.144	0.351	0.046	0.210
Agree	0.424	0.494	0.621	0.486
Strongly agree	0.288	0.453	0.190	0.393

High income				
Strongly disagree	0.099	0.298	0.133	0.340
Disagree	0.323	0.468	0.461	0.499
Neither agree nor disagree	0.288	0.453	0.179	0.384
Agree	0.228	0.420	0.192	0.395
Strongly agree	0.062	0.241	0.035	0.185
Advancement opportunities				
Strongly disagree	0.116	0.321	0.149	0.357
Disagree	0.324	0.468	0.466	0.499
Neither nor	0.267	0.443	0.195	0.397
Agree	0.231	0.422	0.165	0.372
Strongly agree	0.060	0.238	0.024	0.154
Interesting work				
Strongly disagree	0.026	0.158	0.098	0.297
Disagree	0.085	0.280	0.282	0.451
Neither agree nor disagree	0.173	0.378	0.263	0.441
Agree	0.467	0.499	0.304	0.460
Strongly agree	0.249	0.432	0.054	0.227
Work independently				
Strongly disagree	0.044	0.205	0.049	0.216
Disagree	0.118	0.323	0.163	0.337
Neither nor	0.136	0.343	0.130	0.337
Agree	0.454	0.498	0.542	0.499
Strongly agree	0.248	0.432	0.117	0.321
Useful to society				
Strongly disagree	0.019	0.136	0.008	0.090
Disagree	0.066	0.248	0.054	0.227
Neither nor	0.174	0.379	0.168	0.374
Agree	0.446	0.497	0.585	0.493
Strongly agree	0.296	0.456	0.184	0.388
Help others				
Strongly disagree	0.026	0.160	0.022	0.146
Disagree	0.086	0.280	0.100	0.301
Neither nor	0.159	0.365	0.195	0.397
Agree	0.450	0.497	0.550	0.498
Strongly agree	0.280	0.449	0.133	0.340
Contact with people				

Strongly disagree	0.011	0.105	0.011	0.095
Disagree	0.036	0.187	0.054	0.213
Neither nor	0.076	0.264	0.122	0.331
Agree	0.447	0.497	0.645	0.479
Strongly agree	0.430	0.495	0.168	0.374
**Work expectations**				
Job security	0.741	1.183	0.520	1.108
High income	1.245	1.318	1.805	1.283
Advancement opportunities	1.113	1.253	1.474	1.186
Interesting work	0.577	1.029	1.065	1.205
Work independently	0.214	1.181	0.301	1.130
Useful to society	0.059	1.014	0.195	0.869
Help others	0.088	1.042	0.220	0.980
Contact with people	-0.316	1.027	-0.062	0.914

Based on 2015 ISSP data. [a] The entire sample combines the full sample and China, the number of observations is 17,938. [b] The sample size of China is 369.
