# Supplementary material for: Why are Chinese workers so unhappy? A comparative cross-national analysis of job satisfaction, job expectations, and job attributes
Source: PLoS One. 2019 Sep 26;14(9):e0222715. doi: 10.1371/journal.pone.0222715 (PMC6762101; doi:10.1371/journal.pone.0222715)
Supplement: S2 Table — (PDF) [file pone.0222715.s005.pdf]

S2 Table. Summary statistics for the three clusters

	Cluster 1	Cluster 2	Cluster 3
Hours worked weekly	42.06	37.56	38.33
Monthly income in USD	657.06	5403.83	2959.06
Age	41.41	43.89	43.72
Years of education	12.74	14.48	13.88
Family size	3.36	2.89	2.83
Marital status	2.03	2.04	2.03

Country-specific average values based on 2015 ISSP data.
