# Supplementary material for: Why are Chinese workers so unhappy? A comparative cross-national analysis of job satisfaction, job expectations, and job attributes
Source: PLoS One. 2019 Sep 26;14(9):e0222715. doi: 10.1371/journal.pone.0222715 (PMC6762101; doi:10.1371/journal.pone.0222715)
Supplement: S3 Table — (PDF) [file pone.0222715.s006.pdf]

S3 Table. Job satisfaction regressions

	(1)	(2)	(3)[a]
Australia	-0.458***	-0.214**	-0.083
	(0.093)	(0.086)	(0.077)
Austria	0.430***	0.431***	0.350***
	(0.057)	(0.050)	(0.049)
Belgium	-0.186***	0.105**	0.154***
	(0.051)	(0.045)	(0.043)
Chile	0.133*	0.358***	0.545***
	(0.080)	(0.078)	(0.083)
China	-0.667***	-0.445***	-0.188***
	(0.062)	(0.063)	(0.061)
Taiwan (province of China)	-0.210***	-0.178***	-0.070
	(0.051)	(0.047)	(0.047)
Croatia	-0.171***	0.099	0.269***
	(0.064)	(0.061)	(0.058)
Czech Republic	-0.169***	0.061	0.179***
	(0.061)	(0.056)	(0.053)
Denmark	-0.025	0.143**	0.069
	(0.062)	(0.056)	(0.0523)
Estonia	-0.259***	-0.082*	0.063
	(0.053)	(0.049)	(0.048)
Finland	-0.055	0.150***	0.194***
	(0.061)	(0.055)	(0.053)
France	-0.376***	-0.023	0.126**
	(0.067)	(0.060)	(0.059)
Georgia	-0.278***	-0.251***	-0.052
	(0.081)	(0.082)	(0.083)
Hungary	-0.312***	-0.141*	-0.025
	(0.077)	(0.072)	(0.071)
Iceland	-0.037	0.060	0.219***
	(0.062)	(0.056)	(0.052)
India	-0.286***	-0.113	0.275*
	(0.106)	(0.123)	(0.152)
Israel	0.045	0.113*	0.186***
	(0.061)	(0.058)	(0.059)
Japan	-1.107***	-0.632***	-0.498***
	(0.062)	(0.055)	(0.054)
Latvia	-0.0512	0.010	0.172***
	(0.067)	(0.061)	(0.058)
Lithuania	-0.475***	-0.271***	-0.022
	(0.061)	(0.058)	(0.060)

Mexico	0.250***	0.349***	0.513***
	(0.067)	(0.066)	(0.066)
New Zealand	-0.216***	-0.079	-0.005
	(0.075)	(0.079)	(0.073)
Norway	-0.030	0.130***	0.145***
	(0.050)	(0.045)	(0.045)
Philippines	0.189**	0.228**	0.255***
	(0.096)	(0.093)	(0.091)
Poland	-0.599***	-0.251***	0.161***
	(0.062)	(0.054)	(0.057)
Russia	-0.194***	0.143***	0.217***
	(0.055)	(0.053)	(0.052)
Slovakia	-0.245***	-0.024	0.177***
	(0.063)	(0.056)	(0.056)
Slovenia	-0.156**	0.196***	0.162***
	(0.066)	(0.059)	(0.057)
South Africa	-0.216**	-0.094	0.071
	(0.086)	(0.079)	(0.071)
Spain	0.052	0.177***	0.391***
	(0.061)	(0.056)	(0.053)
Suriname	0.046	0.117*	0.173***
	(0.063)	(0.063)	(0.067)
Sweden	-0.231***	-0.033	-0.009
	(0.061)	(0.053)	(0.050)
Switzerland	0.275***	0.293***	0.244***
	(0.053)	(0.050)	(0.047)
Great Britain	-0.201***	-0.077	-0.004
	(0.062)	(0.056)	(0.051)
United States	-0.041	0.115**	0.205***
	(0.061)	(0.055)	(0.052)
Male	-0.021	-0.039**	-0.054***
	(0.018)	(0.017)	(0.016)
Married	0.151***	0.137***	0.108***
	(0.025)	(0.022)	(0.022)
Divorced	0.084**	0.117***	0.120***
	(0.037)	(0.033)	(0.032)
Widowed	0.141**	0.103**	0.074
	(0.060)	(0.051)	(0.049)
Age	-0.039***	-0.026***	-0.021***
	(0.005)	(0.005)	(0.004)
Age^2	0.050***	0.033***	0.027***
	(0.005)	(0.005)	(0.005)
Education	0.013	0.0004	0.017

	(0.015)	(0.013)	(0.014)
Education^2	2.80e-05	0.0002	-0.0007
	(0.001)	(0.0005)	(0.0005)
Family size	-0.001	-0.004	-0.009
	(0.007)	(0.007)	(0.006)
Income: middle	-0.141***	-0.091***	-0.053**
	(0.028)	(0.026)	(0.026)
Income: high	0.057**	0.065**	0.031
	(0.029)	(0.027)	(0.027)
Work hours		0.002*	0.0002
		(0.0008)	(0.0008)
Work pressure (never)		0.175***	0.156***
		(0.022)	(0.021)
Work pressure (often)		-0.155***	-0.128***
		(0.019)	(0.019)
Work pressure (always)		-0.367***	-0.284***
		(0.032)	(0.031)
Work time conditions (can't decide)		-0.022	0.012
		(0.019)	(0.018)
Work time conditions (free to decide)		0.055	0.033
		(0.038)	(0.036)
Work schedule (can't decide)		-0.109***	-0.066**
		(0.034)	(0.033)
Work schedule (free to decide)		-0.024	0.00242
		(0.023)	(0.023)
Daily work organization (can't decide)		-0.193***	-0.114***
		(0.021)	(0.020)
Daily work organization (free to decide)		0.186***	0.173***
		(0.022)	(0.021)
Employer-employee (relation bad)		-0.501***	-0.413***
		(0.044)	(0.043)
Employer-employee (good)		0.601***	0.496***
		(0.022)	(0.022)
Relation between colleagues (bad)		-0.248***	-0.211***
		(0.078)	(0.0715)
Relation between colleagues (good)		0.302***	0.246***
		(0.028)	(0.029)
Δ job security			-0.062***
			(0.013)
Δ high income			-0.090***
			(0.012)
Δ advancement opportunities			-0.019*
			(0.012)

Δ work independently			0.002
			(0.013)
Δ interesting job			-0.286***
			(0.016)
Δ useful to society			-0.010
			(0.015)
Δ contact with people			0.041***
			(0.01)
Δ help others			0.009
			(0.014)
Constant	5.879***	5.016***	5.190***
	(0.145)	(0.142)	(0.149)
Observations	20,517	19,191	17,938
R squared	0.073	0.263	0.357

Based on 2015 ISSP data. The dependent variable is job satisfaction measured on a 7-point scale. [a] Includes dummy variables that have a value equal to one if the expectations gap is negative or zero, and zero otherwise. Standard errors in parentheses. * p<0.1, ** p<0.05, *** p<0.01.
